# Variability in the anatoxin gene clusters of *Cuspidothrix issatschenkoi* from Germany, New Zealand, China and Japan

**DOI:** 10.1371/journal.pone.0200774

**Published:** 2018-07-19

**Authors:** Andreas Ballot, Pia I. Scherer, Susanna A. Wood

**Affiliations:** 1 Norwegian Institute for Water Research, Oslo, Norway; 2 Limnological Research Station Iffeldorf, Technical University of Munich, Munich, Germany; 3 Cawthron Institute, Private Bag 2, Nelson, New Zealand; National Cheng Kung University, TAIWAN

## Abstract

Anatoxin-a and homoanatoxin-a are neurotoxic cyanotoxins produced by benthic and planktonic cyanobacteria worldwide. These toxins are produced by the cyanobacterial genera *Dolichospermum*, *Cuspidothrix*, *Phormidium*, *Oscillatoria*, *Tychonema* and *Cylindrospermum*. In the present study the *ana* gene clusters (*anaA*-*anaG*; c. 21.1 kilobases) of two anatoxin producing *Cuspidothrix issatschenkoi* strains from Germany: (NIVA-CYA 711) and New Zealand (CAWBG02) were sequenced and compared with the *ana* gene clusters of two *C*. *issatschenkoi* strains from Japan (RM-6 and LBRI48) and one from China (CHABD3). All five *ana* gene clusters are characterized by the same gene order for *anaA-anaG*. Similarities were highest (99.56–99.57%) between German (NIVA-CYA 711), New Zealand (CAWBG02) and Chinese (CHABD3) strains. Similarities were lower (91.40–91.67%) when compared to the Japanese strains (RM-6 and LBRI48). Collectively, 2,037 variable sites (328 single nucleotide polymorphisms and 9 insertions/deletions, comprising 1,709 nucleotides) were found in the *ana* gene clusters of the German, New Zealand and Japanese strains compared to the Chinese strain (CHABD3). The *ana* gene clusters of the German (NIVA-CYA 711), New Zealand (CAWBG02) and Japanese (RM-6 and LBRI48) strains were characterized by 83, 84, 255 and 231 SNP’s compared to the Chinese strain (CHABD3), respectively. The *anaE* and *anaF* genes showed the highest variability in all five strains and are recommended as the best genetic markers for further phylogenetic studies of the *ana* gene cluster from *C*. *issatschenkoi*.

## Introduction

Toxin producing cyanobacteria are found worldwide in terrestrial and aquatic environments [1]. Most research to date has focused on the most frequently found producers of the hepatotoxic microcystins. Although initially thought to be a rarely produced toxin, an increasing number of species are now known to produce the neurotoxin anatoxin-a (ANTX) and its homologue homoanatoxin-a (HANTX). Producers have been identified from planktonic and benthic environments [2–5]. ANTX and HANTX production is described from heterocytous cyanobacteria e.g. *Dolichospermum flos-aquae* (Brébisson ex Bornet & Flahault) P.Wacklin, L.Hoffmann & J.Komárek, *Cuspidothrix issatschenkoi* (Usachev) P.Rajaniemi, Komárek, R.Willame, P. Hrouzek, K.Kastovská, L.Hoffmann & K.Sivonen and from non-heterocytous cyanobacteria e.g. *Phormidium autumnale* Gomont, *Phormidium favosum* Gomont, *Oscillatoria* sp. and *Tychonema* sp. [4, 6–11]. ANTXs have been detected in many freshwater habitats including ponds, lakes and rivers and their presence has been associated with a number of animal poisoning incidents, including birds, cattle and dogs [2, 9, 12].

*Cuspidothrix issatschenkoi* is a filamentous nostocalean cyanobacterium which occurs sporadically in the phytoplankton of mesotrophic and eutrophic standing waters in the northern temperate zone, although it rarely forms blooms [13]. In contrast, Wood et al. [8] describe dense blooms of this species in the southern temperate zone in New Zealand. This species has been shown to produce ANTX and HANTX (China, Germany, Japan and New Zealand) and saxitoxins (STX) (Portugal); [7, 8, 14, 15]. Filaments of *C*. *issatschenkoi* lacking heterocytes have been confused with *Raphidiopsis meditteranea* Skuja but later confirmed to belong to the genus *Cuspidothrix* by genetic analyses [8, 14, 16, 17].

The first *ana* gene cluster was analyzed in *Oscillatoria* sp. (strain PCC 6506), a cyanobacterium which produces both ANTX and HANTX [11]. A study by Rantala-Ylinen et al. [18] revealed the complete *ana* gene cluster in *Anabaena* sp. 37 a planktonic strain originally isolated from Finnish Lake Sääskjärvi. Other published *ana* gene clusters are from *Anabaena* sp. WA102 (CP011456) and *Cylindrospermum stagnale* PC7417 (CP003642.1) [19, 20].

In all *ana* gene clusters sequenced to date *anaB* to *anaG* are arranged in the same order, whereas *anaA*, *anaI*, *anaJ* and *anaH* are characterized by different species dependent arrangements [21]. Recently, the partial *ana* gene cluster (*anaA-anaG* gene) from two *C*. *issatschenkoi* strains isolated from Lake Biwa, Japan and one isolated from Xinghu Lake, China was sequenced [14]. It is unclear if *anaI*, *anaJ* and *anaH* are also present in these strains. No information about the *ana* gene cluster of *C*. *issatschenkoi* strain CAWBG02 from New Zealand is available and only a 434 base pair (bp) long segment of the *anaF* gene has been described for the German strain NIVA-CYA 711 (former SP33) [7].

The putative biosynthesis pathway encoded by the *ana* gene cluster is described by Mann et al. [22] and Mejean et al. [21]. In a first step the cyclic amino acid proline is activated and attached by an adenylation protein AnaC to AnaD, an acyl carrier protein (ACP). AnaB then oxidizes the prolyl-ACP to 1-pyrroline-5-carboxyl-ACP (P5CACP). In a following step the polyketide synthase (PKS) AnaE adds a fully reduced acetate unit to P5CACP. An acetate unit is then added by another PKS AnaF, followed by a Michael type cyclization leading to an anatoxinic thioester linked to the ACP domain. The PKS AnaG supposedly adds an acetate unit without reduction and performs a methylation. This results in the homoanatoxin precursor. Without methylation the product of this last reaction is the precursor for ANTX [21].

The aim of this study was to sequence the entire *ana* gene clusters (*anaA*-*anaG*) from two ANTX-producing *C*. *issatschenkoi* strains from New Zealand and Germany. This will significantly increase the knowledge on phylogenetic relationships and intraspecific variations in the *ana* gene clusters of this species.

## Material and methods

### Genomic DNA extraction, PCR amplification and sequencing

Genomic DNA was isolated according to Ballot et al. [23].

Primers to amplify the *ana* gene cluster of *C*. *issatschenkoi* strains NIVA-CYA 711 (Germany) (former SP33 [7]) maintained at the Norwegian culture collection NORCCA at the Norwegian Institute for Water Research and CAWBG02 (New Zealand) [8] were designed using anatoxin synthesis gene cluster sequences KM245023, KM245024 and KM245025 of *C*. *issatschenkoi* strains LBRI48, RM-6 (from Japan) and CHABD3 (from China), respectively [14]. Primers were designed for PCR products covering around 3,500 base pairs (bp) each using the FastPCR software [24, 25] S1 Table. Consecutive sequences were chosen to overlap at around 500 nucleotides. PCR’s for segments of the *ana* gene cluster were performed on a Bio-Rad CFX96 Real-Time PCR Detection System (Bio-Rad Laboratories, Oslo, Norway) using the iProof High-Fidelity PCR Kit (Bio-Rad Laboratories, Oslo, Norway).

*Ana* gene cluster fragments were amplified in separate PCR reactions using the same cycling protocol comprising of: one cycle of 5 min at 94°C, and then 35 cycles each consisting of 10 s at 94°C, 20 s at 62°C, and 20 s at 72°C, followed by a final elongation step of 72°C for 5 min. PCR products were visualized by 1.5% agarose gel electrophoresis with GelRed staining and UV illumination. Amplified PCR products were purified through QIAquick PCR purification columns (QIAGEN, Hilden, Germany), and the DNA was eluted in elution buffer according to the manufacturer's protocol. In the case of gaps or PCR’s not working well, shorter overlapping sequences were amplified and sequenced. After positive amplification intermediate forward and reverse primers were designed with the FastPCR software for the sequencing step [24, 25]. The *ana* gene cluster fragments were sequenced using the primers depicted in S1 Table. For each PCR product, both strands were sequenced on an ABI 3730 Avant genetic analyzer using the BigDye terminator V.3.1 cycle sequencing kit (Applied Biosystems, Thermo Fisher Scientific Oslo, Norway) according to the manufacturer's instructions.

### Nucleotide sequence accession numbers

The sequence data were deposited in the European Nucleotide Archive (ENA) under the following accession numbers: LT984882 (*C*. *issatschenkoi* NIVA-CYA 711; Germany, *ana* gene cluster), LT984883 (*C*. *issatschenkoi* CAWBG02; New Zealand, *ana* gene cluster).

### Phylogenetic analysis

Partial sequences of the *ana* gene cluster in both *C*. *issatschenkoi* strains CAWBG02 (New Zealand) and NIVA-CYA 711 (Germany) were analyzed using the Seqassem software package (Version 07/2008) [26]. The Align MS Windows-based manual sequence alignment editor (Version 03/2007) [26] was used to obtain DNA sequence alignments, which were then corrected manually.

Phylogenetic trees based on partial 16S rRNA gene sequences obtained from GenBank and the *ana* gene cluster (*anaA-anaG*) and on the *anaF* gene were constructed using the Maximum Likelihood (ML) method in Mega V7 [27]. The calculation of the 16S rRNA gene phylogenetic tree was based on 1,063 bp. K2+G was found to be the best fitting model for the 16S rRNA gene. *Dolichospermum flos-aquae* (HG970731) was used as outgroup. The tree for the *ana* gene cluster was calculated without an outgroup, because no complete *ana* gene cluster sequence of other species fitted to the *ana* gene cluster of *C*. *issatschenkoi*. The *anaF* genes of *Anabaena* sp. 37 (JF803645), *Anabaena* sp. WA102 (CP011456), *Oscillatoria* sp. PCC6506 (FJ477836) and *Cylindrospermum stagnale* PCC7417 (CP003642) and the homologue sequence of non ANTX-producing *Moorea producens* (former *Lyngbya majuscula*) PAL-8-15-08-1 (CP017599) were included in the tree for the *anaF* gene. HKY+G was found to be the best fitting model for the *ana* gene cluster and GTR+G for the *anaF* gene. All ML analyses were performed with 1,000 bootstrap replicates using Mega V.7 [27].

### ELISA

*Cuspidothrix issatschenkoi* strains CAWBG02 (New Zealand) and NIVA-CYA 711 (Germany) were retested for ANTX production by using the Abraxis anatoxin ELISA kit (Abraxis LLC,Warminister, PA, USA) following the manufacturer’s instructions. The aim of this was to confirm that they were still producing ANTX despite been in cultivation for a considerable period of time. The test is a direct competitive ELISA based on the recognition of ANTX by a monoclonal antibody. Anatoxin standards varied between 0.15 and 5.0 ppb. Before analysis, 5 mL of culture material from each cyanobacterial strain was frozen and thawed three times to extract the toxins. The ELISA results do not distinguish between dissolved and cell-bound toxins, and in this study we only interpret the results qualitatively to assess whether ANTX are still been produced. The color reaction of the ELISA test was evaluated at 450 nm on a Perkin Elmer 1420 Multilabel counter Victor3 (Perkin Elmer, Waltham, USA).

## Results

### Phylogenetic characterization

**Fig 1:**
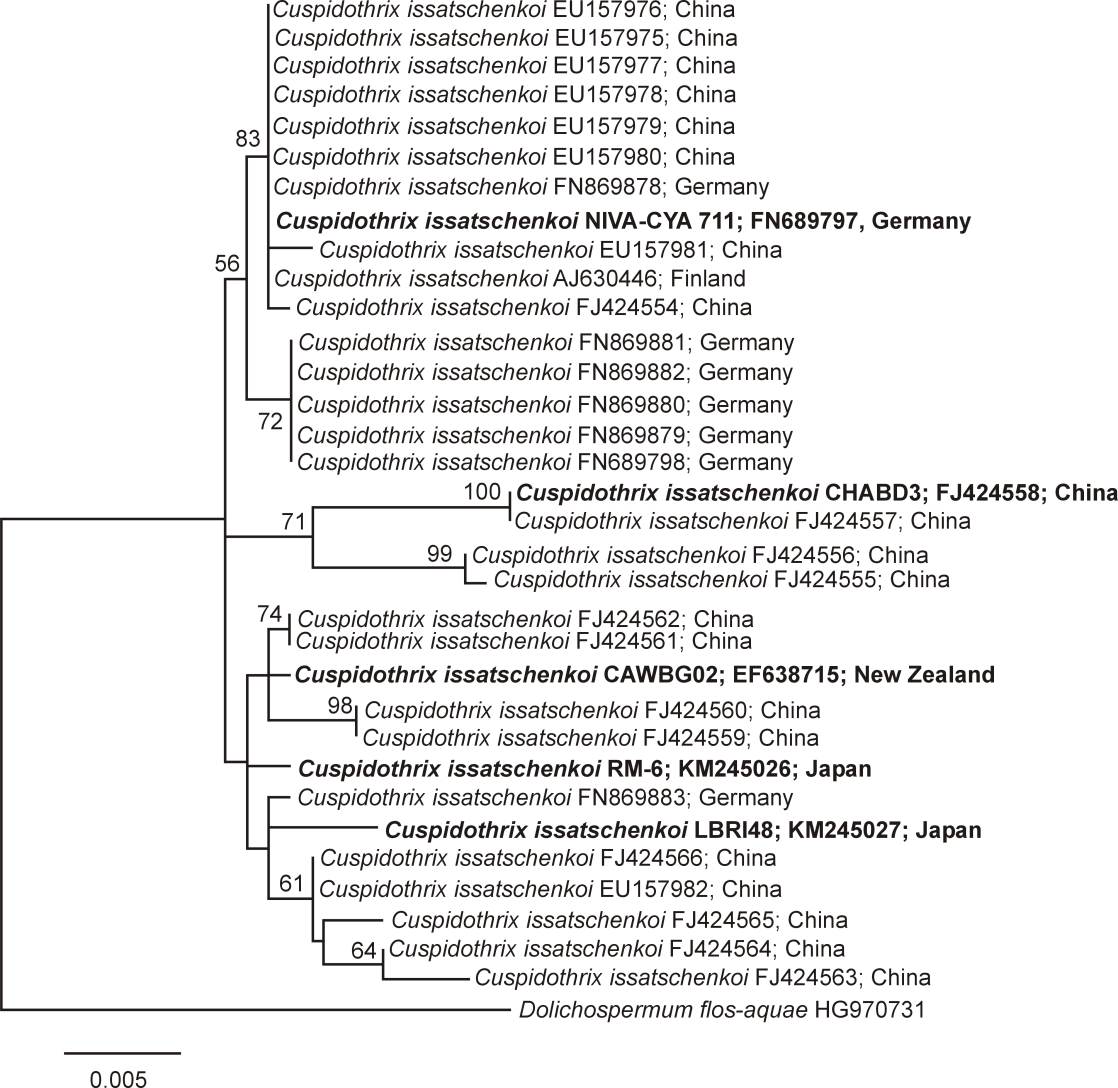
Maximum Likelihood tree of the 16S rRNA gene of *Cuspidothrix issatschenkoi*. Strains from this study are marked in bold. Bootstrap values above 50 are included. The scale bar indicates 0.5% sequence divergence.

The 16S rRNA gene analysis of the four known ANTX-producing and the one non ANTX-producing *C*. *issatschenkoi* strains, showed that CAWBG02 (New Zealand), NIVA-CYA711 (Germany) and CHABD3 (China) are located in separate sub-clusters. Both Japanese strains RM-6 and LBRI48 clustered together with CAWBG02 (New Zealand; Fig 1). Sequence similarity was highest (99.81%) between the strains CAWBG02 (New Zealand) and LBRI48 (Japan), whereas similarity was slightly lower (99.53%) for CAWBG02 (New Zealand) and NIVA-CYA 711 (Germany; Table 1). Lower similarities (98.59% or 98.68%) were identified between NIVA-CYA 711 (Germany) and CHABD3 (China), or CAWBG02 (New Zealand) and CHABD3 (China), respectively (Table 1). The lowest similarity (98.12%) was between CHABD3 (China) and RM-6 (Japan).

**Table 1:** Similarities of the 16S rRNA gene based on 1,063 base pairs for *Cuspidothrix issatschenkoi* strain NIVA-CYA 711 (FN689797; Germany), CAWBG02 (EF638715; New Zealand), CHABD3 (FJ424558; China), LBRI48 (KM245026; Japan) and RM-6 (KM245027; Japan). The values in the upper right triangle are the percent identities between a pair of sequences. The values in the lower left triangle are the numbers of dissimilar nucleotides.

	CHABD3	CAWBG02	NIVA-CYA 711	RM-6	LBRI48
	(China)	(New Zealand)	(Germany)	(Japan)	(Japan)
CHABD3 (China)	-	98.68	98.59	98.12	98.49
CAWBG02 (New Zealand)	14	-	99.53	99.44	99.81
NIVA-CYA 711 (Germany)	15	5	-	99.15	99.53
RM-6 (Japan)	20	6	9	-	99.44
LBRI48 (Japan)	16	2	5	6	-

The *ana* gene clusters (*anaA*-*anaG*) of strains NIVA-CYA 711 (Germany) and CAWBG02 (New Zealand) were characterized by high similarity (99.56–99.57%) to the cluster sequences of CHABD3 (China). Relatively low similarities (c. 91.40–91.67%) were identified between the three strains NIVA-CYA 711 (Germany), CAWBG02 (New Zealand) and CHABD3 (China) and strains RM-6 and LBRI48 (Japan; Table 2).

**Table 2:** Similarities of the *ana* gene cluster (*anaA-anaG*) based on 22,802 base pairs for *Cuspidothrix issatschenko*i strain NIVA-CYA 711 (LT984882; Germany), CAWBG02 (LT984883; New Zealand), CHABD3 (KM245025; China): LBRI48 (KM245023; Japan) and RM-6 (KM245024; Japan). The values in the upper right triangle are the percent identities between a pair of sequences. The values in the lower left triangle are the numbers of dissimilar nucleotides.

	CHABD3	CAWBG02	NIVA-CYA 711	RM-6	LBRI48
	(China)	(New Zealand)	(Germany)	(Japan)	(Japan)
CHABD3 (China)	-	99.57	99.56	91.40	91.51
CAWBG02 (New Zealand)	91	-	99.8	91.56	91.66
NIVA-CYA 711 (Germany)	92	43	-	91.57	91.67
RM-6 (Japan)	1961	1924	1921	-	99.59
LBRI48 (Japan)	1936	1901	1898	93	-

**Fig 2:**
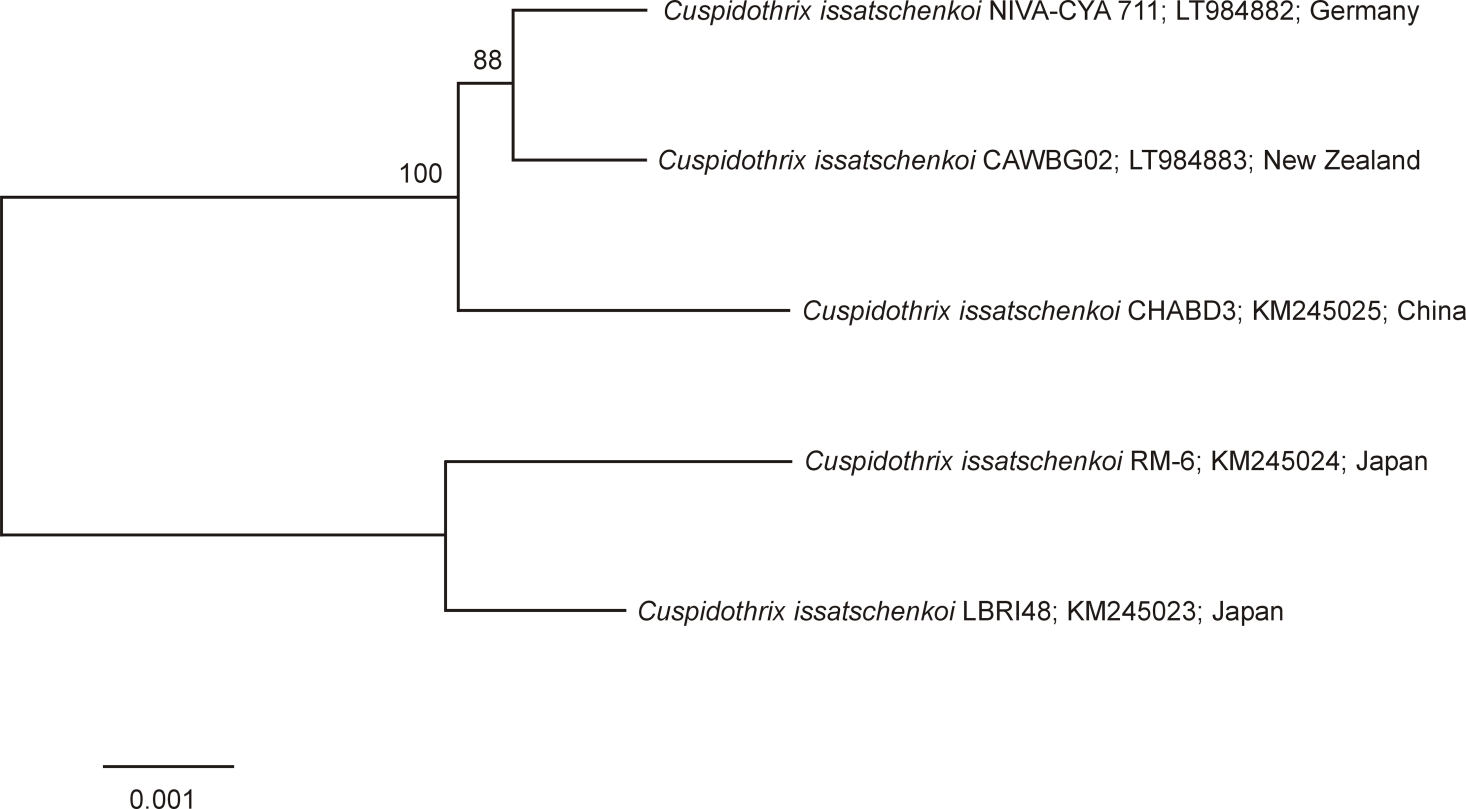
Maximum Likelihood tree based on the *ana* gene clusters of five *Cuspidothrix issatschenkoi* strains included in this study. Bootstrap values above 50 are included. The scale bar indicates 0.1% sequence divergence.

**Fig 3:**
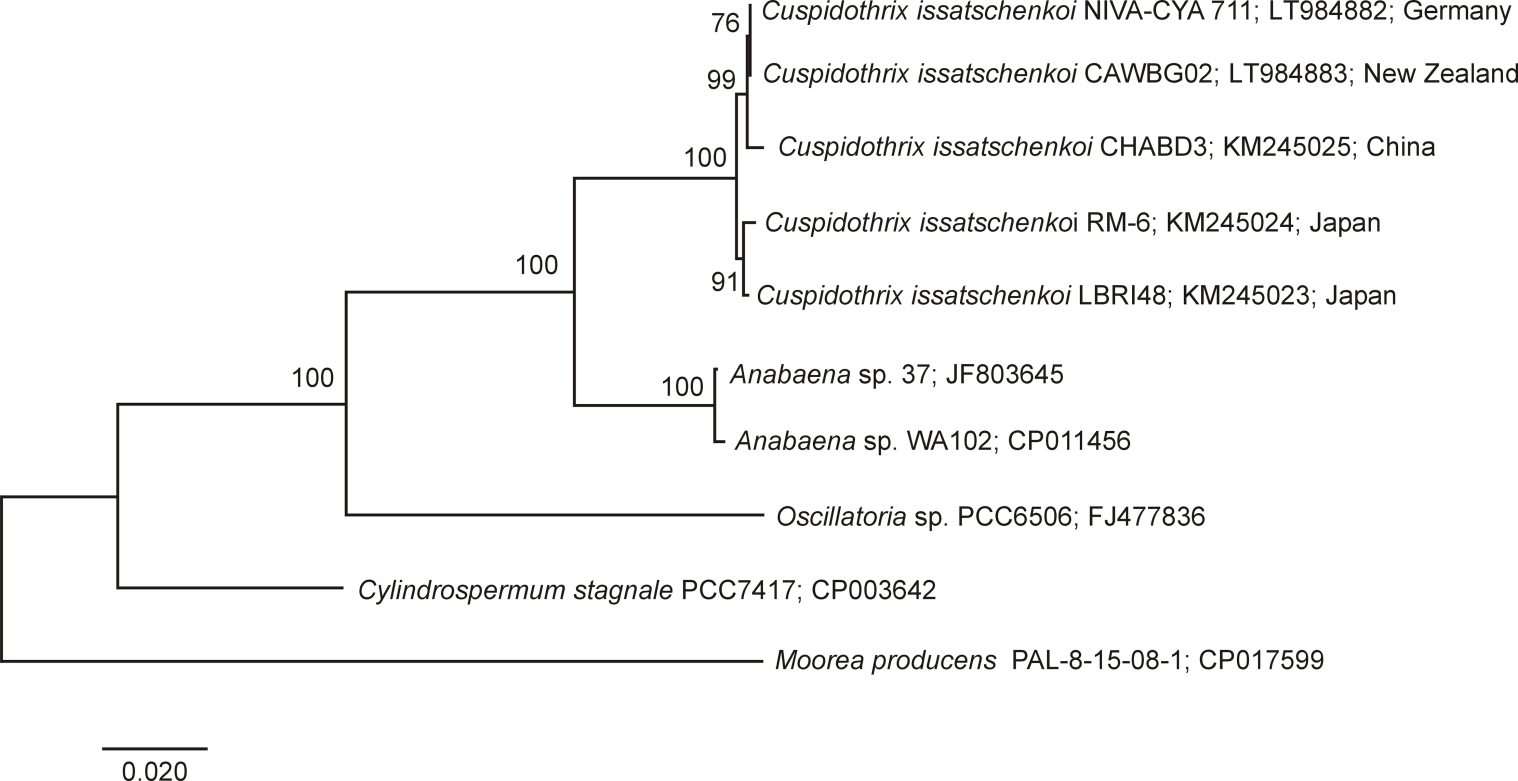
Maximum Likelihood tree based on the *anaF* gene clusters of the five *Cuspidothrix issatschenkoi* strains included in this study. *anaF* genes of *Anabaena* sp., *Oscillatoria* sp., *Cylindrospermum stagnale* are included in the tree. A homologous sequence of *Moorea producens* is used as outgroup. Bootstrap values above 50 are included. The scale bar indicates 2% sequence divergence.

The ML tree distinguished clearly two separate clusters (Fig 2). The *ana* gene cluster sequence of NIVA-CYA 711 (Germany) was most closely related to CAWBG02 (New Zealand), and both clustered with CHABD3 (China). Strains LBRI48 and RM-6 (Japan) formed a separate sub-cluster. Similar results were achieved by calculating a ML tree based on the *anaF* gene (Fig 3). The order of the genes (*anaA-anaG*) in *ana* gene clusters from NIVA-CYA 711 (Germany) and CAWBG02 (New Zealand) were identical to the three other *C*. *issatschenkoi* strains CHABD3 (China), RM-6 and LBRI48 (Japan) [14]. The gene cluster was comprised of around 21.1 to 22.8 kilobase each and altogether 337 variable sites (328 single nucleotide polymorphisms (SNP) and 9 insertions/deletions (In/Dels) comprising 1,709 bp, were detected when the four *C*. *issatschenkoi* strains were compared using *ana* of strain CHABD3 (China) as a reference (Table 3). All five *ana* gene clusters differed slightly in the number and presence of SNP’s and In/Dels. When compared to CHABD3 (China) *ana* of *C*. *issatschenkoi* strains CAWBG02 (New Zealand), NIVA-CYA 711 (Germany), RM-6 and LBRI48 (Japan) were characterized by the presence of 84, 83, 255 and 231 SNP’s, respectively. Of the 328 SNP’s detected, 250 SNP’s were transversions (Tv) and 78 SNP’s were transitions (Tn) (Table 3). In total, 222 SNP’s were located in the coding regions *anaA*, *orf 1*, *anaB*, *anaC*, *anaD*, *anaF* and *anaG*. One hundred and thirty-four of the 222 SNP’s were synonymous and 88 were nonsynonymous substitutions. One hundred and six SNP’s were located in non-coding regions (Table 1). In coding areas of *ana*, RM-6 (Japan) showed the highest ratio of 1 SNP per 125 bp, while NIVA-CYA 711 (Germany) showed the lowest ratio with 1 SNP per 299 bp. In non-coding areas, the highest ratio of 1 SNP per 20 bp was observed in RM-6 (Japan), while the lowest ratio was found in CAWBG02 (New Zealand) and NIVA-CYA 711 (Germany) with 1 SNP per 78 bp. Compared to strain CHABD3 (China) deletions were found in the coding *anaF* gene (three bp in CAWBG02 (New Zealand), NIVA-CYA 711 (Germany), RM-6 and LBRI48 (Japan) and further six bp in RM6 and LBRI48 (Japan)) and in the noncoding areas between the *anaA* and *orf1* genes (one bp in RM-6 and LBRI48 (Japan)) and between the *orf1* and *anaB* genes (one bp in RM-6 and LBRI48 (Japan) and further six bp in CAWBG02 (New Zealand), NIVA-CYA 711 (Germany), RM-6 and LBRI48 (Japan)), respectively. Insertions were found in the coding *anaG* gene (1686 bp in RM-6 and LBRI48 (Japan)) and in the non-coding areas between the *anaA* and *orf1* genes (RM-6 (Japan), one bp) and between the *orF1* and *anaB* genes (two bp in RM-6 and one bp in LBRI48 (Japan)).

**Table 3:** Genes and non-coding regions in the *ana* gene cluster with single nucleotide polymorphisms (SNP) compared to *Cuspidothrix issatschenkoi* strain CHABD3 from China. Tn = Transition, Tv = Transversion, In = Insertion, Del = Deletion. CAWBG02 (New Zealand), NIVA-CYA 711 (Germany), RM-6 (Japan), LBRI48 (Japan).

Gene	Tn	Tv	In/Del	Nonsyn	Total	Strain (SNP, InDel, Non-syn) compared to *Cuspidothrix issatschenkoi* strain CHABD3			
*anaA*	30	10	0	24	40	CAWBG02 (5, - ,1);	NIVA-CYA 711 (7, - ,2);	RM-6 (33, - ,23);	LBRI48 (29, - ,22)
*orf1*	16	4	0	9	20	CAWBG02 (2, - ,1);	NIVA-CYA 711 (4, - ,1);	RM-6 (18, - ,8);	LBRI48 (18, -, 8)
*anaB*	6	1	0	2	7	CAWBG02 (2, - ,1);	NIVA-CYA 711 (2, - ,1);	RM-6 (4, -, -);	LBRI48 (1, -, -)
*anaC*	12	2	0	5	14	CAWBG02 (3, - ,1);	NIVA-CYA 711 (4, - ,1);	RM-6 (12, - ,4);	LBRI48 (4, - ,2)
*anaD*	7	1	0	0	8	CAWBG02 (7, -, -);	NIVA-CYA 711 (4, -, -);	RM-6 (3, -, -);	LBRI48 (4, -, -)
*anaE*	36	9	0	18	45	CAWBG02 (19, - ,13);	NIVA-CYA 711 (17, - ,12);	RM-6 (31, - ,13);	LBRI48 (35,- ,14)
*anaF*	41	16	9	18	66	CAWBG02 (22, 3 ,7);	NIVA-CYA 711 (21, 3 ,7);	RM-6 (49, 9 ,15);	LBRI48 (43, 9, 13)
*anaG*	26	5	1,686	12	1,717	CAWBG02 (7, -, 5);	NIVA-CYA 711 (7, -, 5);	RM-6 (19 ,1686 ,10);	LBRI48 (27, 1686, 10)
**Subtotal coding**	**174**	**48**	**1,695**	**88**	**1,917**				
*anaA-orf1*	28	15	5	0	48	CAWBG02 (2, -, -);	NIVA-CYA 711 (1, -, -);	RM-6 (41, 5, -);	LBRI48 (29, 4, -)
*orf1-anaB*	45	14	9	0	68	CAWBG02 (12, 6, -);	NIVA-CYA 711 (15, 6, -);	RM-6 (43, 8, -);	LBRI48 (39, 9, -)
*anaB-anaC*	0	0	0	0	0	CAWBG02 (-, -, -);	NIVA-CYA 711 (-, -, -);	RM-6 (-, -, -);	LBRI48 (-, -, -)
*anaC-anaD*	0	0	0	0	0	CAWBG02 (-, -, -);	NIVA-CYA 711 (-, -, -);	RM-6 (-, -, -);	LBRI48 (-, -, -)
*anaD-anaE*	3	0	0	0	3	CAWBG02 (3, -, -);	NIVA-CYA 711 (1, -, -);	RM-6 (1, -, -);	LBRI48 (1, -, -)
*anaE-anaF*	0	0	0	0	0	CAWBG02 (-, -, -);	NIVA-CYA 711 (-, -, -);	RM-6 (-, -, -);	LBRI48 (-, -, -)
*anaF-anaG*	0	1	0	0	1	CAWBG02 (-, -, -);	NIVA-CYA 711 (-, -, -);	RM-6 (1,-, -);	LBRI48 (1, -, -)
**Subtotal noncoding**	**76**	**30**	**14**	**0**	**120**				
**Total**	**250**	**78**	**1,709**	**88**	**2,037**				

The *orf1* was the same length in CAWBG02 (New Zealand), NIVA-CYA 711 (Germany) and CHABD3 (China). In the two strains RM-6 and LBRI48 (Japan) it was 363 bp shorter. CAWBG02 (New Zealand) differed with 2 bp and NIVA-CYA711 with 4 bp from CHABD3 (China; Table 3).

### ELISA

Both strains *C*. *issatschenkoi* strains CAWBG02 (New Zealand) and NIVA-CYA 711 (Germany) tested positive for ANTX using ELISA with values clearly exceeding the upper standard value of 5 ppb. The ELISA was used only as a qualitative control to test that the strains were still able to produce ANTX.

## Discussion

The two ANTX-producing *C*. *issatschenkoi* strains from Germany (NIVA-CYA 711) and New Zealand (CAWBG02) possess *ana* gene clusters which are in the same gene order as in the non ANTX-producing *C*. *issatschenkoi* strain from China (CHABD3) and the ANTX-producing strains from Japan (RM-6 and LBRI48) [14]. The *ana* gene clusters of three strains from Germany, China and New Zealand were highly conserved (> 99.5%). The similarity of the *ana* gene cluster of the two Japanese strains (RM-6 and LBRI48) to these strains was much lower, suggesting a different evolution of their *ana* gene cluster. This is also reflected in the ML tree of the complete *ana* gene cluster where the New Zealand (CAWBG02) and German (NIVA-CYA 711) strains form one sub-cluster together with the Chinese strain (CHABD3) while the two Japanese strains (RM-6 and LBRI48) are grouped in a separate sub-cluster. High similarities above 94% have also been described for the *sxt* gene clusters of PSP toxin producing *Aphanizomenon gracile* strains from different geographical regions [23]. Horizontal gene transfer mediated by plasmids has been suggested to be involved in the evolutionary process of the *ana* gene cluster [28, 29]. However, according to Dittmann et al. [29], there is no clear evidence that horizontal gene transfer explains the sporadic distribution of the *ana* gene cluster. The marine and non ANTX-producing *M*. *producens* possesses a number of genes with a high similarity to those in the known *ana* gene clusters. This fact and the future discovery of more unknown taxa possessing *ana* gene clusters or similar clusters will assist in enhancing knowledge on the unknown evolutionary origin of this toxin gene cluster.

Interestingly, the 16S rRNA gene analysis did not show the same pattern and better reflects the geographic distribution of the five *C*. *issatschenkoi* strains.

### Insertions and deletions

The New Zealand (CAWBG02) and German (NIVA-CYA 711) strains do not possess the insertion of 1,686 bp which is present in the *anaG* gene in the Japanese strains (RM-6 and LBRI48). The 562 amino acids encoded by this segment contain a methyl transferase [14] which is believed to be a prerequisite for the production of HANTX. The production of HANTX has been verified in the Japanese strains (RM-6 and LBRI48) [14, 16].

The New Zealand (CAWBG02), German (NIVA-CYA 711) and Japanese (RM-6 and LBRI48) strains are characterized by a deletion of three bp causing the lack of the amino acid serine (amino acid position 696) in the *anaF* gene compared to the Chinese strain (CHABD3). Serine is, however, present at the same location in the *anaF* gene of the ANTX producers *Anabaena* sp. 37, *Anabaena* sp. WA102, *Cylindrospermum stagnale* PCC 7417 and *Oscillatoria* sp. PCC 6506. These strains lack the amino acid leucine at amino acid position 698. As only non ANTX-producing Chinese (CHABD3) strain possesses both amino acids, it suggests that it is not the type of amino acid but the additional amino acid at this position that could have caused a change in the function of the PKS AnaF, leading to the lack of ANTX production in the Chinese strain (CHABD3). The Japanese strains (RM-6 and LBRI48) lack a further six bp in the *anaF* gene causing the absence of valine, serine and glutamic acid in the encoded polyketide synthase. This, however, does not seem to affect the function of this PKS as the Japanese strains (RM-6 and LBRI48) still produce ANTX. ANTX-producing *C*. *issatschenkoi* strains NIVA-CYA 711 and CAWBG02, *Anabaena* sp. 37 and *Anabaena* sp. WA102 do possess the same three amino acids at this location.

The *anaF* genes of ANTX producers *Anabaena* sp. 37, *Anabaena* sp. WA102, *Cylindrospermum stagnale* PCC 7417 and *Oscillatoria* sp. PCC 6506 are characterized by relatively low similarities (84.9 to 86.7%). This is much lower than between non ANTX-producing Chinese (CHABD3) and ANTX-producing strains from New Zealand (CAWBG02), Germany (NIVA-CYA 711) and Japan (RM-6 or LBRI48). This raises the question as to which SNP’s are responsible for the lack of ANTX production in the Chinese strain (CHABD3). According to Mann et al. [22] AnaF adds an acetate unit and catalyzes a Mannich-type cyclization step to form anatoxic acid.

Further deletions and insertions have been found in the noncoding areas *anaF*-*orf1* and *orf1*-*anaB*. Whether those have caused the lack of ANTX production in the Chinese strain (CHABD3) is unknown.

As observed in in the Chinese strain (CHABD3) the *orf1* gene in the New Zealand (CAWBG02) and German (NIVA-CYA 711) strains is not truncated as described for both Japanese strains (RM-6 and LBRI48) [14]. *Orf1* encodes a cyclase which is hypothesized to catalyse the bicyclic structure formed by Claisen-type cyclization of the ANTX molecule [14]. This truncation does not appear to affect ANTX or HANTX production.

Interestingly, the marine cyanobacterium *M*. *producens* which is not known to produce ANTX [30] possesses a homologous PKS encoding gene with around 77 to 81% similarity to the *anaF* gene. The species is known to be a producer of many bioactive substances [31]. A blast search revealed that with the exception of *orf1* the genome of *M*. *producens* strain Pal -8-15-08-1 contains genes or sequences similar to *anaA-anaG* in the *ana* gene cluster of all ANTX producers. The sequences are characterized by at least 79 to 88% similarity to those described from the *ana* gene cluster found in the ANTX and non ANTX-producing *C*. *issatschenkoi* strains and other ANTX producers. This suggests that a common ancestor possessed a homologous gene cluster or homologous genes which are involved in different biosynthetic pathways.

### Single nucleotide polymorphisms

ANTX production is lacking in the Chinese strain (CHABD3) [14]. This is most likely due to the numerous SNP’s and point mutations. Several studies have pointed out that the loss of toxin production can be caused by point mutations in otherwise intact toxin encoding gene clusters or its regulatory genes [32, 33].

Compared to the Chinese strain (CHABD3), the German strain (NIVA-CYA 711) is characterized by the lowest number of SNP’s, while one of the Japanese strains (RM-6) has the highest. On average a single non-coding SNP can be expected in at least 200±500 base pairs of non-coding DNA, and a single coding SNP in 500±1000 base pairs of coding DNA [34]. The investigated strains in this study are characterized by much higher SNP ratios of between 1 SNP per 125 bp (Japan; RM-6) and 1 SNP per 299 bp (Germany; NIVA-CYA 711) in coding areas, indicating relatively low sequence conservation for the anatoxin biosynthesis machinery in *C*. *issatschenkoi*. Much lower values of 1 SNP per 1178 bp—2062 bp have been found in coding areas in a similar study on the PSP toxin encoding gene cluster in *A*. *gracile* strains from Germany, Spain and USA [23].

Even the lowest ratio of one SNP per 78 bp (New Zealand; CAWBG02 and Germany; NIVA-CYA 711) in non-coding areas of the ANTX encoding gene cluster is much higher than the ratio for those areas described by Brumfield et al. [34]. Their study was, however, conducted on eukaryotic organisms.

The relatively high SNP ratios in *ana* of the investigated *C*. *issatschenkoi* strains suggest relatively fast evolutionary changes in its genes. It is unclear how fast these changes have occurred in the single strains of *C*. *issatschenkoi*. Recent studies on bacterial genomes have shown that in different bacterial species the estimated evolutionary rates spanned several orders of magnitude, from approximately 10−^5^ to 10^−8^ nucleotide substitutions per site year ^-1^ [35].

Compared to reference strain CHABD3 (China) only 10 of the 88 observed nonsynonymous SNP’s are present in *ana* of all four ANTX-producing *C*. *issatschenkoi* strains or the other known ANTX-producing taxa. As they are found in the *anaC*, *anaE*, *anaF* and *anaG* gene only, one or more changes in these four genes are most likely responsible for the lack of ANTX production in the Chinese strain (CHABD3). The one nonsynonymous SNP and its following change of amino acid could cause significant changes in protein structure, function and stability [36]. However, further studies on other ANTX-producing strains and non-producing strains possessing *ana* are necessary to distinguish the responsible nonsynonymous SNP’s from the ones without any effect.

Compared to the Chinese strain (CHABD3), in the New Zealand (CAWBG02) and German (NIVA-CYA 711) strains more than 57% of the SNP’s are located in the *anaE* and *anaF* gene only and in the Japanese strains (RM-6 and LBRI48) more than 41%. This indicates both genes as suitable markers for further phylogenetic studies on ANTX-producing *C*. *issatschenkoi*. *AnaE* which is characterized by the highest number of nonsynonymous substitutions in all four strains, compared to the Chinese strain (CHABD3), encodes a polyketide synthase. Eleven of the 18 nonsynonymous substitutions present in the *anaE* gene are found in the German (NIVA-CYA 711), New Zealand (CAWBG02) and Japanese (RM-6 and LBRI48) strains. The PKS AnaE receives the (S)-1-pyrroline-5-carboxyl-AnaD (P5C-AnaD) for elongation [22]. The PKS encoding *anaF* gene is characterized by between 7 and 15 nonsynonymous substitutions in the German (NIVA-CYA 711), New Zealand (CAWBG02) and Japanese (RM-6 and LBRI48) strains compared to the one isolated from China (CHABD3). AnaF adds then another acetate unit and catalyzes a Mannich-type cyclization step to form anatoxic acid linked to the ACP module of AnaF [22]. It is possible that the presence of glutamic acid and glycine in AnaF of Chinese strain (CHABD3) instead of glutamin and serine or arginine present in AnaF of all known ANTX-producing strains has caused a change in the function and lead to the inactivity of this PKS. Glycine is a hydrophobic amino acid, while serine and arginine are polar or charged amino acids. Glutamate is a polar and glutamic acid a charged amino acid. The exchange of these amino acids may therefore change the chemical properties of the whole protein. Further studies are required to confirm this.

In contrast to the New Zealand (CAWBG02) and German (NIVA-CYA 711) strains the Japanese strains (RM-6 and LBRI48) possess also a conspicuously high number of SNP’s in the *anaA*, *orf1* and *anaG* genes while *anaD* is the only gene in the investigated *ana* gene cluster of *C*. *issatschenkoi* where no nonsynonymous substitutions were found in all strains.

## Conclusions

The intraspecific investigation of two *ana* gene clusters in ANTX-producing *C*. *issatschenkoi* strains from Germany and New Zealand and one non toxin-producing strain from China has shown that gene conservation in the *ana* gene cluster is high among these strains, but relatively low compared to two ANTX-producing strains from Japan. The observed variability in the numbers and positions of both SNP’s and insertions and deletions in the *ana* gene clusters highlight important differences between strains. In this study all four ANTX-producing and one non ANTX-producing but *ana* possessing *C*. *issatschenkoi* strains were investigated. Further studies encompassing strains from a wider geographic area will be necessary to determine the distribution of ANTX-producing *C issatschenkoi* strains and genetic diversity among gene clusters. As *C*. *issatschenkoi* is only sporadically found in temperate water bodies it may be difficult to increase knowledge about the geographic distribution of such strains. The highest intraspecific genetic variability was observed in the *anaE* and *anaF* genes and these are therefore recommended for further phylogenetic research of *C*. *issatschenkoi*.

## Supporting information

S1 Table Primers for PCR and sequencing of the ana gene cluster of Cuspidothrix issatschenkoi. (PDF)
