# Supplementary material for: Variability in the anatoxin gene clusters of Cuspidothrix issatschenkoi from Germany, New Zealand, China and Japan
Source: PLoS One. 2018 Jul 19;13(7):e0200774. doi: 10.1371/journal.pone.0200774 (PMC6053186; doi:10.1371/journal.pone.0200774)
Supplement: S1 Table — (PDF) [file pone.0200774.s001.pdf]

Table S1. Primers for PCR and sequencing of the *ana* gene cluster of *Cuspidothrix issatschenkoi*

Segment	**Primer**	**Use**	**primer sequence**	**genes covered**
1	ana3f	PCR, sequencing	actcagaaggatgtttcca	*anaA orf1, anaB* partial
	ana527f	sequencing	gcaatgagcgctcccatactg	
	ana1022f	sequencing	gttacagtaagcactgtcaaa	
	ana1582f	sequencing	agaacaagcacaggcagta	
	ana2268f	sequencing	tacttrgmgatagggttgc	
	ana2792f	sequencing	tggtattcacggatggcct	
	ana3089r	sequencing	tccatgttttcgagccgtt	
	ana2513r	sequencing	aggacttacccattgacagg	
	ana1991r	sequencing	caaattttgcgttgccyagag	
	ana1485r	sequencing	taaccaagggcatctttgc	
	ana937r	sequencing	tatcaatgggctacaggct	
	ana470r	sequencing	tctcaggtcrcgctgctccta	
	ana3471r	PCR, sequencing	tgaccaaattgcttgtgggt	
2	ana3006f	PCR, sequencing	ggttggattgcatcccacg	*anaB* partial, *anaC, anaD, anaE* partial
	ana6477r	PCR, sequencing	tgacagaaaccgaccgcta	
	ana3522f	sequencing	ttgcggttggagaaygcca	
	ana4026f	sequencing	ttgaagagcggtgccaccta	
	ana4553f	sequencing	agccaaattcattgccgcag	
	ana5019f	sequencing	atttaccgcaccggggatctt	
	ana5531f	sequencing	aggattgtygactcaatgg	
	ana6059f	sequencing	tttcgcatggcatcacgagaa	
	ana5998r	sequencing	aatcttacccggcatagga	
	ana5486r	sequencing	gatcgtatgccaactcttgg	
	ana4984r	sequencing	ggaacccktacttgagcagtc	
	ana4455r	sequencing	gaaatggcgcatgactgga	
	ana3947r	sequencing	actgctcaaggtatgtgcca	
	ana3453r	sequencing	tgaccaaattgcttgtgggt	
3	ana6001f	PCR, sequencing	tacacgggctggaggtttctt	*anaE* partial*,*
	ana9489r	PCR, sequencing	gctaaaccagtgcggtctg	
	ana6524f	sequencing	tgatggattcgtccaaggag	
	ana7038f	sequencing	aaccaaagctggcaggtgtt	
	ana7561f	sequencing	actttatcccgaacaaccag	
	ana8040	sequencing	cgttcgctcaagtcgctgg	
	ana8613f	sequencing	cagaacaccgcgctttcggtc	
	ana9031f	sequencing	gcattctgaggggaaagccct	
	ana8997r	sequencing	tagaccattcctcgggtgg	
	ana8427r	sequencing	acctcaacccaacagcgtt	
	ana7963r	sequencing	tttcagttcggctacaatgg	
	ana7471r	sequencing	ttcgcgacccatrtcaacg	
	ana6990r	sequencing	ttatgccaaggaatacgcga	
	ana6516r	sequencing	aaggttttgcagcgaccat	

Table S1 cont. Primers for PCR and sequencing of the *ana* gene cluster of *Cuspidothrix issatschenkoi*

Segment	**Primer**	**Use**	**primer sequence**	**genes covered**
4	ana9023f	PCR, sequencing	cgcttgtggcattctgagg	*anaE* partial*, anaF partial,*
	ana12467r	PCR, sequencing	cggctagacatctttccgggt	
	ana9519	sequencing	gtggacagcgaccacacctc	
	ana10026f	sequencing	caagcatcgctcctgtagc	
	ana10532f	sequencing	ttagcgcaactgaagggagc	
	ana11054f	sequencing	tacctaattgcgggaggat	
	ana11521f	sequencing	gcgatcacaaggtttgcca	
	ana11990f	sequencing	gtcgaacattccttgcgctc	
	ana12016r	sequencing	tagagttgagcgcaaggaa	
	ana11538r	sequencing	ggcaaaccttgtgatcgccga	
	ana10998r	sequencing	ttaccaatgtgcttggctt	
	ana10515r	sequencing	cctacacctccagccgcat	
	ana9994r	sequencing	agcgayataccgtacccca	
	ana9489r	sequencing	gctaaaccagtgcggtctg	
5	ana12086f	PCR, sequencing	atgagtgtccagcaaacag	*anaE* partial, *anaF* partial,
	ana15418r	PCR, sequencing	gccaaatctagcggtgctt	
	ana12563f	sequencing	ccttgacccacagcagaggct	
	ana13137f	sequencing	gtacctagcggaccttctc	
	ana13630f	sequencing	tgctyaccctcagygccaa	
	ana14143f	sequencing	ttgaaccaagcgcagtcatcg	
	ana14618f	sequencing	agatcatgtcctcaaaccag	
	ana15077f	sequencing	agcagatcatrctctctacga	
	ana15024r	sequencing	ttttcagagccgcactccgaa	
	ana14513r	sequencing	gcgaaaatctggcagcattgg	
	ana13993r	sequencing	atttcctggcagcgctcta	
	ana13468r	sequencing	ttgatcggtagctsctccca	
	ana13024r	sequencing	caaccttcactgcgaacat	
	ana12543r	sequencing	agatcccaaagaagtgcgcgt	
6	ana15002f	PCR, sequencing	aattcggagtgcggctctgaa	*anaF* partial, *anaG* partial
	ana18450r	PCR, sequencing	gccaaacgtagtcattrgcac	
	ana15520f	sequencing	agaagttgtggcgktctcagg	
	ana16036f	sequencing	gttcyctagccgatatgctc	
	ana16505f	sequencing	aatcgaaatgctgctccaa	
	ana17052f	sequencing	gttataggtgcgctaggtc	
	ana17598f	sequencing	agcttaggatgtcgcttgc	
	ana18082f	sequencing	tgagttgtcgcttggcagaa	
	ana18074r	sequencing	aatggcgatcggttcgcgt	
	ana17485r	sequencing	acttgagattggacgtgagct	
	ana17002r	sequencing	gtcagtttgtgcaagttcc	
	ana16445	sequencing	aacaatcacgcgrgctagtcc	
	ana15977	sequencing	attcgccttagcgaaggacga	

Table S1 cont. Primers for PCR and sequencing of the *ana* gene cluster of *Cuspidothrix issatschenkoi*

Segment	**Primer**	**Use**	**primer sequence**	**genes covered**
	ana15499	sequencing	ggaagttaggcccatactc	
7	ana17598f	PCR, sequencing	agcttaggatgtcgcttgc	*anaF* partial, *anaG* partial
	ana20530r	PCR, sequencing	aacgtcggtagccatgttcca	
	ana18023f	sequencing	acaatccgaaattgagcga	
	ana18549f	sequencing	acaggtccttgtctcgccgt	
	ana19033f	sequencing	tcaaggtcttccgccaacg	
	ana20018f	sequencing	aaaactgccggatagcgga	
	ana20017r	sequencing	tgcatcaactgcccyctgg	
	ana19521r	sequencing	ggaaatgagtccgccgagta	
	ana19057r	sequencing	tcttcccgttggcggaagac	
	ana18521	sequencing	gctataggcgttccccgatcc	
8	ana20000f	PCR, sequencing	agarccaaccgtagtcatgg	*anaG* partial
	ana21300r	PCR, sequencing	aatcagcaacgccggataytg	
	ana21147	sequencing	tggaacatggctaccgacgtt	
	ana20530r	sequencing	aacgtcggtagccatgttcca	
